# Exploring the performances of the vibrating barriers for the seismic protection of the Zoser pyramid

**DOI:** 10.1038/s41598-022-09444-x

**Published:** 2022-04-01

**Authors:** P. Cacciola, M. Shadlou, A. Ayoub, Y. F. Rashed, A. Tombari

**Affiliations:** 1 School of Architecture, Technology and Engineering, University of Brighton, Brighton, UK; 2 School of Mathematics, Computer Science and Engineering, City University of London, London, UK; 3 Department of Structural Engineering, Cairo University, Giza, Egypt

**Keywords:** Civil engineering, Natural hazards

## Abstract

In this paper we aim to investigate the use of the Vibrating Barrier (ViBa) as a potential strategy to mitigate the effects of the seismic action on the Zoser Pyramid. The Vibrating Barrier is a structure buried in the soil that is able to absorb a significant portion of the dynamic energy arising from the ground motion. The working principle exploits the dynamic interaction among vibrating structures resting on a compliant semi-infinite space, namely the structure–soil–structure interaction. A reliable numerical simulation of the Zoser Pyramid and the surrounding soil undergoing stochastic ground motion excitations representing the seismicity in Saqqara is presented. Due to the unique structural form, the ViBa is herein optimized through an *ad-hoc* procedure to minimize a response strain energy spectral density used as a synthetic performance parameter. Various layouts of the ViBa have been considered and presented in the paper. The efficiency of the ViBa is assessed by numerical simulation of the finite element model of the ViBa-Soil-Pyramid system and by laboratory testing. Results from a pertinent Monte Carlo study show an evident reduction of the stresses in the Pyramid manifesting the feasibility of this novel strategy to protect historic structures from earthquake-induced ground motion. Experimental results on a 1:500 gelatine model of the pyramid and the surrounding area highlighted the efficiency and efficacy of the proposed approach.

## Introduction

Egypt has an extensive cultural heritage, reaching back more than five thousand years, with a plethora of archaeological sites considered among the most important in the world. The archaeological site of Saqqara, located about 20 km from Cairo city, is appraised as the world's most extensive burial ground with monuments of almost every period of ancient Egyptian civilization. In this area, the Step Pyramid of Djoser or Zoser pyramid is situated. It is therefore an important legacy of ancient construction to be preserved.

Although Egypt is a region of small to moderate magnitude earthquakes^1^, several events that occurred in the area from the late 1980s, with two main events, i.e., the 1992 Cairo earthquake and the 1995 Gulf of Aqaba earthquake, partly damaged the Zoser pyramid. The Pyramid endured an important restoration activity to avoid the risk of collapse leading to its closure to visitors for nearly 14 years^1^. Therefore, it becomes evident that the preservation and conservation of archaeological monuments and sites require unconventional and exceptional seismic analysis and design. Conventional seismic protection systems, mainly based on strengthening techniques or local devices such as dampers or seismic isolators, are based on structural invasive interventions whose application clearly might risk compromising the historical value of the heritage structures such as the Pyramid. Those techniques are generally applied to heritage structures as a repairing technique (as done to the Zoser pyramid) rather than as a preventive strategy to mitigate future seismic actions. Ancient monuments, therefore, are left generally unprotected from future catastrophic events due to the difficulty to protect them without altering their historical value.

**Figure 1:**
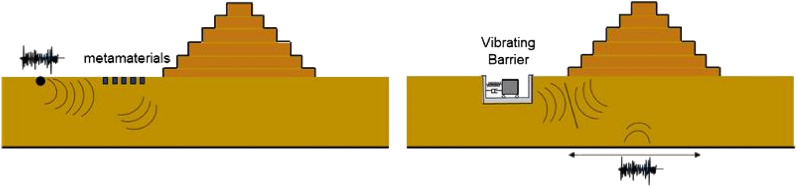
Schematic representation of the working principles of two modern non-local seismic protection systems; in panel (**a**) the seismic metamaterials able to scatter the Rayleigh waves and in panel (**b**), the vibrating barrier technology exploiting the mutual interaction between the device and the structure to be protected.

Therefore, as the traditional localized solutions might become impractical or of difficult applications for ancient structures, alternative non-invasive solutions need to be pursued. One possible strategy dating back to 1968 (see e.g. Woods^2^) is to screen surface waves through trenches or sheet-pile walls in the soil^3^. More recently, a more advanced filtering strategy based on Bragg’s scattering law has led to the development of the concept of seismic metamaterials. Meseguer^4^ et al. conducted experiments on the attenuation of surface-elastic waves in a marble quarry by drilling cylindrical holes in two different configurations. Brûlé et al.^5^ firstly introduced the concept of seismic metamaterials through the means of a regular mesh of cylindrical and empty boreholes showing the effectiveness of an engineered grid in molding surface waves. Finocchio et al.^6^ developed the seismic metamaterials for body waves as a chain of mass-in-mass systems able to filter the S-waves of an earthquake. Colombi et al.^7^ showed that a forest can act as a natural seismic metamaterial by screening Rayleigh waves. The concept has been then extended to the resonant metawedge^8^. Palermo et al.^9^ proposed an engineered metabarrier realized by burying sub-wavelength resonant structures under the soil surface to screen surface waves. The idea has been developed^10^ considering multi-mass resonant units. A study on a large-scale application of seismic metamaterials has been performed by Miniaci et al.^11,12^ showing the effectiveness of this strategy to protect structures from surface waves. Laboratory experiments on metamaterials have been conducted by Colombi^13^ et al. highlighting that the metabarrier could present a viable solution for containing ground borne vibrations, usually confined at the surface while for seismic excitations, where energy is characterized by a very heterogeneous azimuthal distribution, metabarrier might not be an ideal solution and further research is required. Recently, an active approach, adopting the typical terminology in vibration control, has been also proposed (Herbut^14–16^) to mold surface waves through the use of wave generators. The list of references herein reported is certainly not exhaustive, but it aims to highlight the growing body of literature in this direction. It has to be emphasized that the seismic metamaterials concept originates from electromagnetism and optics, hence the approach generally adopted in the studies cited earlier is based on the elastic wave propagation on soil media and how the engineered filter is able to mold the incoming waves to potentially cloak the structure to be protected. An opposite strategy of non-local seismic protection, and therefore not identifiable with the generally know seismic metamaterial, introduced by Cacciola and Tombari^17^, exploits the interaction induced by shear body waves through the soil, between a vibrating device called Vibrating Barrier (ViBa) and every surrounding structure to protect. The Vibrating Barrier (ViBa) is in essence a vibrating spring-mass-damper system, hosted in the soil, which is able to modify the dynamic behaviour of the adjacent structures with the aim to mitigate their seismic responses. It clearly shares the idea of the most recent seismic metamaterials defined as buried-mass resonators, but with the key difference that the ViBa is designed to exploit the structure-soil-structure phenomenon, studied since the early 70s^18,19^. Apart from the origin of the theory behind the development of the mathematical formulation of the ViBa, the structure-soil-structure mechanism highlights the key difference with the seismic metamaterials concept: the structure to be protected and the device are not two separate entities but they are coupled. Therefore, the seismic protection through a vibrating mass in the soil is not accomplished in cascade by screening the incoming wave (as done by using metamaterials) but it is achieved by controlling their mutual interaction. Figure 1 shows schematically these two different approaches.

This key feature gave the opportunity to successfully apply the Vibrating Barrier concept to various case studies. Analyses on the efficiency of the ViBa to protect a single building from shear waves are reported in Cacciola and Tombari^17^ and Cacciola et al.^20^ for structures founded on monopile foundation. Tombari et al.^21^ analyzed the sensitivity of the performance of the vibrating barrier to various design parameters for an industrial building and to protect a cluster of buildings^22^. Due to the large masses involved in the traditional ViBa device Cacciola et al. ^23^ developed an inerter-equipped Vibrating Barrier showing that is possible to reduce the mass of the vibrating unit at least by 50% achieving the same level of performance. Pan and Málaga-Chuquitaype^24^ extended this approach to the control of rocking structures using combined inerters and external resonators.

In this paper, we explore the performance of the traditional Vibrating Barrier as a potential strategy to seismic protect the Zoser pyramid. A stochastic ground motion model able to capture the natural variability in Saqqara is developed through a power spectral density function with random parameters. Moreover, to design the Vibrating Barrier device, a sub-structuring method specifically formulated for protecting the Pyramid is proposed; this proposed approach aims to reduce the computational effort during the iterative analyses required by the optimization design process, without the need of performing multiple analyses on the large numerical model composed of Pyramid, ViBa and surrounding soil. The response strain energy spectral density is herein proposed for the first time as a measure of the overall response of the Pyramid to the stochastic ground motion. The success of the ViBa device to the seismic protection of the Pyramid is measured versus the overall reduction of the strain energy and in the consequent reduction of the averaged octahedral shear (i.e. Von Mises) stresses in all the solid elements of the pyramid model.

## Results

### Simulation of the stochastic excitation for the Zoser pyramid

**Figure 2:**
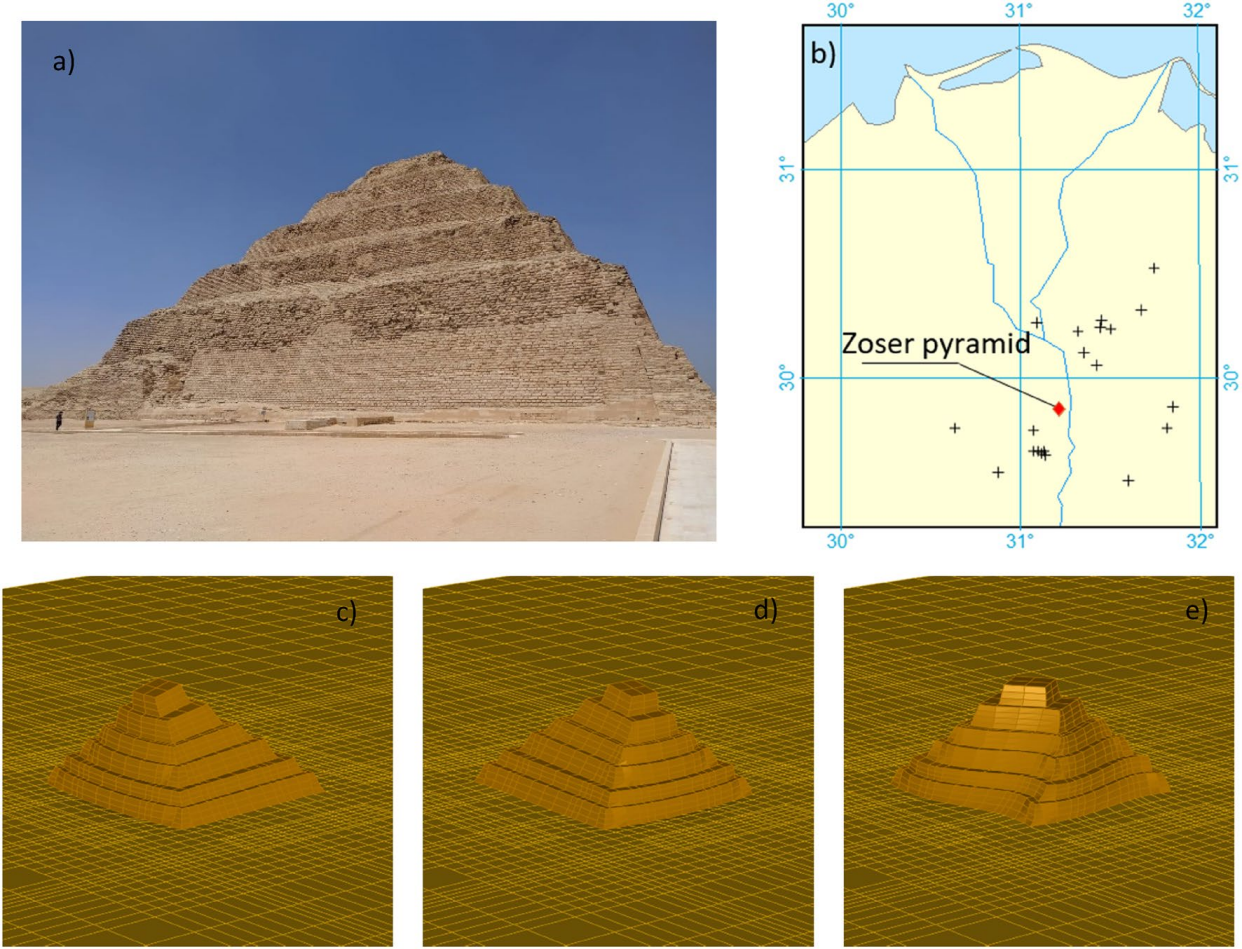
In panel (**a**) photography of the Zoser Pyramid investigated in this study; Panel (**b**) shows the epicentres (marked with “+”) of 24 earthquakes with magnitude M ≥ 3 occurred in and around the Greater Cairo Area and the location of the Zoser pyramid (“diamond”); Panels from (**c**) to (**e**) show the first three modal shapes of the numerical model of the Zoser Pyramid.

The Zoser pyramid (Fig. 2a) is modelled in this study as a solid stepped structure with a rectangular base of about 108 m × 120 m and a total height of about 63 m. Soil investigations and surveying have been supplied by the Cairo University’s team and used to generate the numerical finite element model. Moreover, a soil investigation through a 10-m depth borehole has been undertaken to characterize the soil properties for the Saqqara’s site, determining a limestone/sandy-limestone deposit interspersed with layers of silt. The material properties of Pyramid are considered identical to the soil, modelled as an isotropic linear material defined by the elastic modulus, $E_{p} = 45{\text{ GPa}}$, Poisson's ratio, $\upsilon_{p} = 0.25$ as well as unit density, ${ }\rho_{p}$, of 2900 kg/m^3^. The bedrock, located at 35 m depth, has been determined from previous studies available in the literature^25,26^. The soil stratum between the bedrock and the base of the pyramid is assumed homogeneous with a constant shear wave velocity of 3000 m/sec, derived from the site investigation undertaken in the first 10 m and in agreement with the traditional values of limestone material. A preliminary modal analysis is first carried out for the fixed-base Pyramid model: the first mode, associated with its smallest width (Fig. 2c) has a natural frequency of 10.68 Hz, while the second mode associated with the largest width (Fig. 2d) is characterized by the natural frequency of 10.884 Hz; the third mode (Fig. 2e) which is torsional, occurs at the frequency of 15.03 Hz.

Consequently, a study on the seismic activity in Saqqara relevant to the Zoser pyramid has been carried out. Using the data reported in Reference^27^, the following map (Fig. 2b) of the earthquakes that occurred in the effective seismogenic zone in and around the Cairo Area has been developed. From the distribution of the epicentres (and the distance from the Zoser pyramid), it is clearly expected a large variability of ground motion excitation at the base of the pyramid.

In absence of publicly available seismic records, the Boore method^28^ is adopted to determine the power spectral density function $G_{{\ddot{u}_{g} }} \left( {{\varvec{\alpha}},f,t} \right)$ of the ground acceleration at the bedrock, $\ddot{u}_{g}$, as a function of the vector of random variables ${\varvec{\alpha}}$, the frequency $f$ as well as the time $t$; therefore, an evolutionary power spectral density with random parameters is established. In this paper, we generated the vector of random variables ${\varvec{\alpha}}$ by means of the magnitude, $M_{0}$, epicentral distance $R$, stress drop $\Delta {\upsigma }$, and angle of incidence $\delta$; these random parameters are defined by establishing their probability distribution functions from the seismological data given in literature^27^. An equirectangular approximation is used to determine the distances, $R$, between the coordinates of the epicentres of the past earthquakes and the centre of the Zoser pyramid, as well as the incidence angle $\delta$ with respect to the determined E-W side of the pyramid. Magnitude, $M_{0}$, epicentral distance $R$, and angle of incidence are then considered lognormal distributed. The stress drop ∆σ is taken as uniformly distributed between 15 and 50 bars.

Finally, the proposed adopted mean power spectral density function is given by

1 $$\overline{G}_{{\ddot{u}_{g} }} \left( {f,t} \right) = \mathop \smallint \limits_{{A}}^{{}} G_{{\ddot{u}_g }} \left( {{\varvec{\alpha}},f,t} \right)p_{{A}} \left( {\varvec{\alpha}} \right){\text{ d}}{\varvec{\alpha}}$$

in which the probability density function, $p_{{A}} \left( {\varvec{\alpha}} \right),$ is defined as follows:

2 $$p_{{A}} \left( {\varvec{\alpha}} \right) = p_{M} \left( {M_{0} } \right)p_{R} \left( R \right)p_{S} \left( {\Delta {\upsigma }} \right)p_{\Delta } \left( \delta \right)$$

### Optimal design of the vibrating barrier

**Figure 3:**
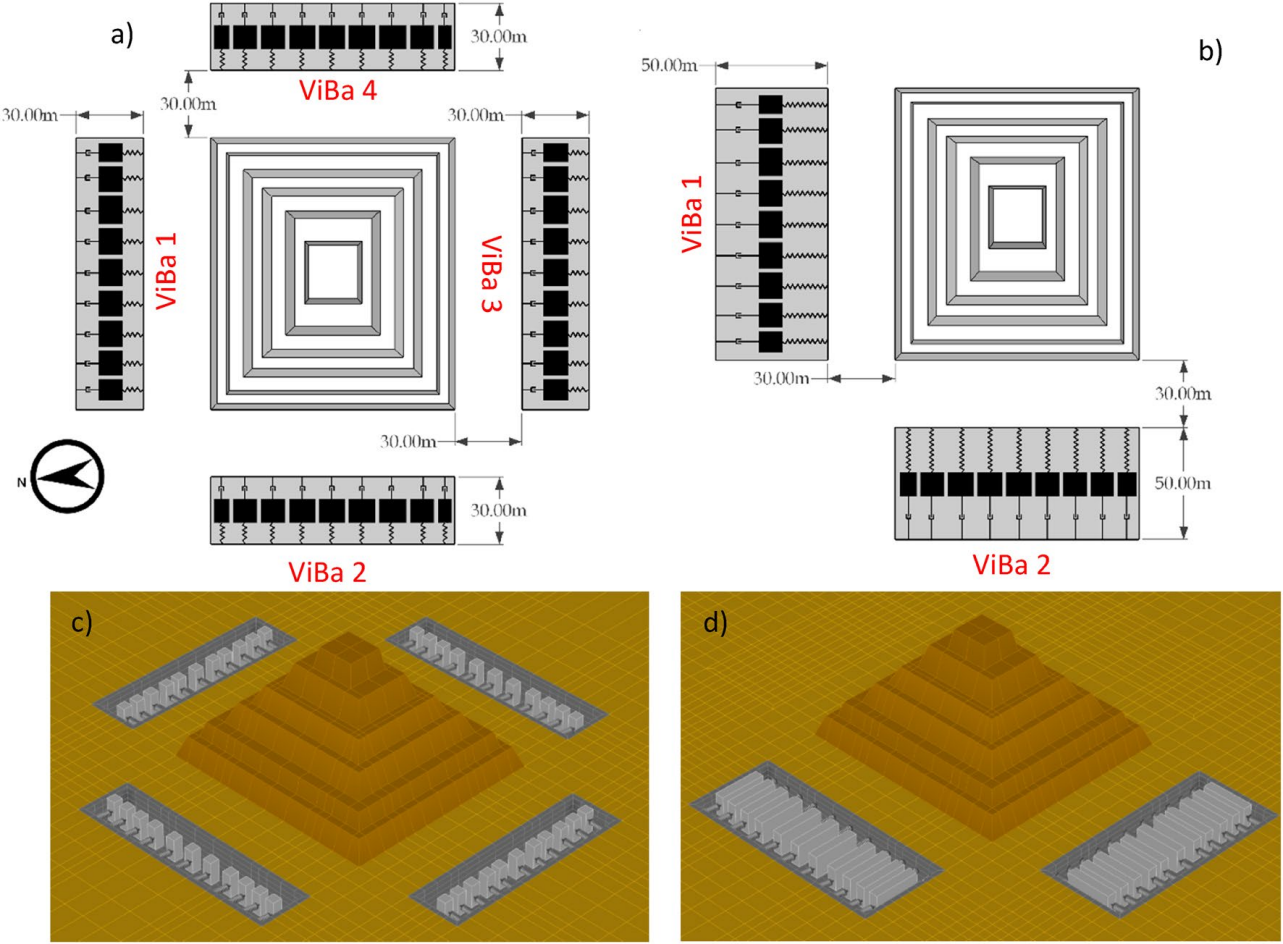
ViBa configurations for Case 1 in panels (**a**) and (**c**) and Case 2 in panels (**b**) and (**d**). Each excavation can include several units that can act asynchronously (with different mechanical properties) or synchronously (identical mechanical properties) according to the specific design. ViBa protects the Pyramid by exploiting the soil-structure interaction phenomenon and not by filtering the seismic waves; therefore, the two configurations will be both effective regardless the direction of the seismic motion.

To mitigate the seismic response of the Zoser Pyramid, the Vibrating Barrier is adopted in this section. A parametric analysis has been conducted considering various layouts able to host steel masses to generate inertial forces proportional to a range between 40 and 80% of the total mass of the pyramid. Figure 3 shows two potential configurations of the Vibrating Barrier to protect the Zoser pyramid. These configurations, named Case 1 and Case 2, have been selected with the objective to minimize the number of excavations and keep a balance between distance and performance of the ViBa. The box foundation of the ViBa is made of four concrete retaining walls and a matt foundation. As the Vibrating Barrier exploits the structure-soil-structure mechanism, the closer the excavation is, the larger will be the interaction and therefore, lower inertial forces should be generated. On the other hand, the use of large excavations too close to the pyramid might have negative implications of practical or heritage nature. Also, it has been decided to use a single slot to accommodate multiple vibrating masses. However, totally independent units would lead to similar results.

For a selected configuration, the problem to design the ViBa is therefore reduced to determine the mechanical parameters of the devices (i.e. spring stiffnesses $K_{V}$ and loss factors $\eta_{V}$) collected in the vector ${\varvec{\beta}}$ so that relevant response quantities are reduced. As the pyramid is not a conventional structure, the selection of the response quantity to be minimized poses additional challenges. Certainly, the selection of the top displacements or Von-Mises stresses in a given point might be attractive due to their simplicity, but it is not, in general, representative of the overall potential risk of damage of the pyramid.

In this paper we propose to minimize the cumulative response strain energy determined in the frequency domain through the strain energy spectral density^29^ ${\Psi }\left( {\text{f}} \right)$ herein defined as:

3 $${\Psi }\left( {{\varvec{\alpha}};{\varvec{\beta}};{f}} \right) = \frac{1}{2}\mathop \smallint \limits_{V}^{{}} {\varvec{s}}^{{\text{T}}} \left( {{\varvec{\alpha}};{\varvec{\beta}};{f}} \right){\varvec{e}}\left( {{\varvec{\alpha}};{\varvec{\beta}};{f}} \right){\text{dV}}$$

where ${\varvec{s}}\left( {f} \right)$ and ${\varvec{e}}\left( {f} \right)$ are the Fourier Transform of the response stress vector ${\varvec{\sigma}}\left( {t} \right)$ and strain vector ${\varvec{e}}\left( {t} \right)$ for each element of the pyramid of volume V. The minimization of the cumulative response strain energy can be directly linked to the minimization of several response parameters such as the peak acceleration, displacement, and Von Mises stress, leading to the seismic protection of the Pyramid from future damages. The purpose of the use of the cumulative response strain energy is to minimize a non-local synthetic parameter representative of the overall risk of damage of the pyramid under random input accounting for different angles of incidence. To evaluate the strain energy spectral density is therefore necessary to: (i) determine all the normal and shear stresses and strains for each element; (ii) calculate the strain energy spectral density for each element and (iii) add each individual contribution to determine the strain energy spectral energy of the whole pyramid. It is noted that the response strain energy of the pyramid is a function of the vector of random variables ${\varvec{\alpha}}$ defining the stochastic ground motion model and also of the vector ${\varvec{\beta}}$ collecting the ViBa mechanical parameters to be determined. The optimization problem of Eq. (3) is performed for the two cases by considering the total mass of the ViBa devices, $M_{V}$, equal to 80% of the Pyramid mass; optimal parameters are reported in Table 1. The approach proposed to reduce the computational demand of the optimization problem is described in the “Method” section.

**Table 1:** Design parameters of each ViBa unit for Case 1 and Case 2 obtained from optimization procedure. For multiple masses in the same excavation, the total mass can be split accordingly.

Case		Mass Ratio	$M_{V}^{{}} \left( {Kg} \right)$	$K_{V}^{{}} \left( {N/m} \right)$	$C_{V}^{{}} \left( {N.s/m} \right)$
1	ViBa 1	0.4	386,183,836	1.628E+12	2.51E+09
	ViBa 2	0.4	386,183,836	1.823E+12	2.65E+09
	ViBa 3	0.4	386,183,836	1.793E+12	2.63E+09
	ViBa 4	0.4	386,183,836	1.689E+12	2.55E+09
2	ViBa 1	0.8	772,367,672	3.862E+12	5.46E+09
	ViBa 2	0.8	772,367,672	3.937E+12	5.51E+09

The optimization problem has yielded to the reduction of 33% and 26.7% of the strain energy spectral density, for Case 1 and Case 2, respectively, with respect to the existing Pyramid. It has to be emphasized that those reduction values refer to the material properties adopted in the model. A detailed discussion on the influence of soil properties and sensitivities of the response of structures protected by the Vibrating Barrier can be found in Literature^20,21^. Also, for practical purposes (i.e. reduced computational demand), it is convenient to assign the ViBa loss factor, $\eta_{V}$, equal to 0.1 (i.e. identical to the rest of the model); under this scenario, the computed reductions are equal to 29.9% and 24.32% for Case 1 and Case 2, respectively. Because of the small differences between the outcomes, the following analyses have been conducted by considering the assigned loss factor.

### Seismic protection of the Zoser pyramid through vibrating barriers

To verify the efficiency of the designed Vibrating Barriers for mitigating the seismic response of the Zoser pyramid, a Monte Carlo Simulation (MCS) is undertaken by considering a set of acceleration ground motion realizations generated through the Boore model described in “Simulation of the stochastic excitation for the Zoser pyramid” section. Modal time-history analyses of the numerical model created in Sect. 2 are performed by using $n$ = 500 generated realizations for each of the considered uniformly distributed subsets of Magnitude, $M_{0}$, specifically $M_{0} = \left[ {2 - 3} \right]$, $M_{0} = \left[ {3 - 5} \right]$ and $M_{0} = \left[ {5 - 6} \right]$, which have been split to achieve faster convergence. The results obtained from the previously described Case 1 and Case 2 are compared to the existing scenario of the Zoser Pyramid with no seismic protection.

**Figure 4:**
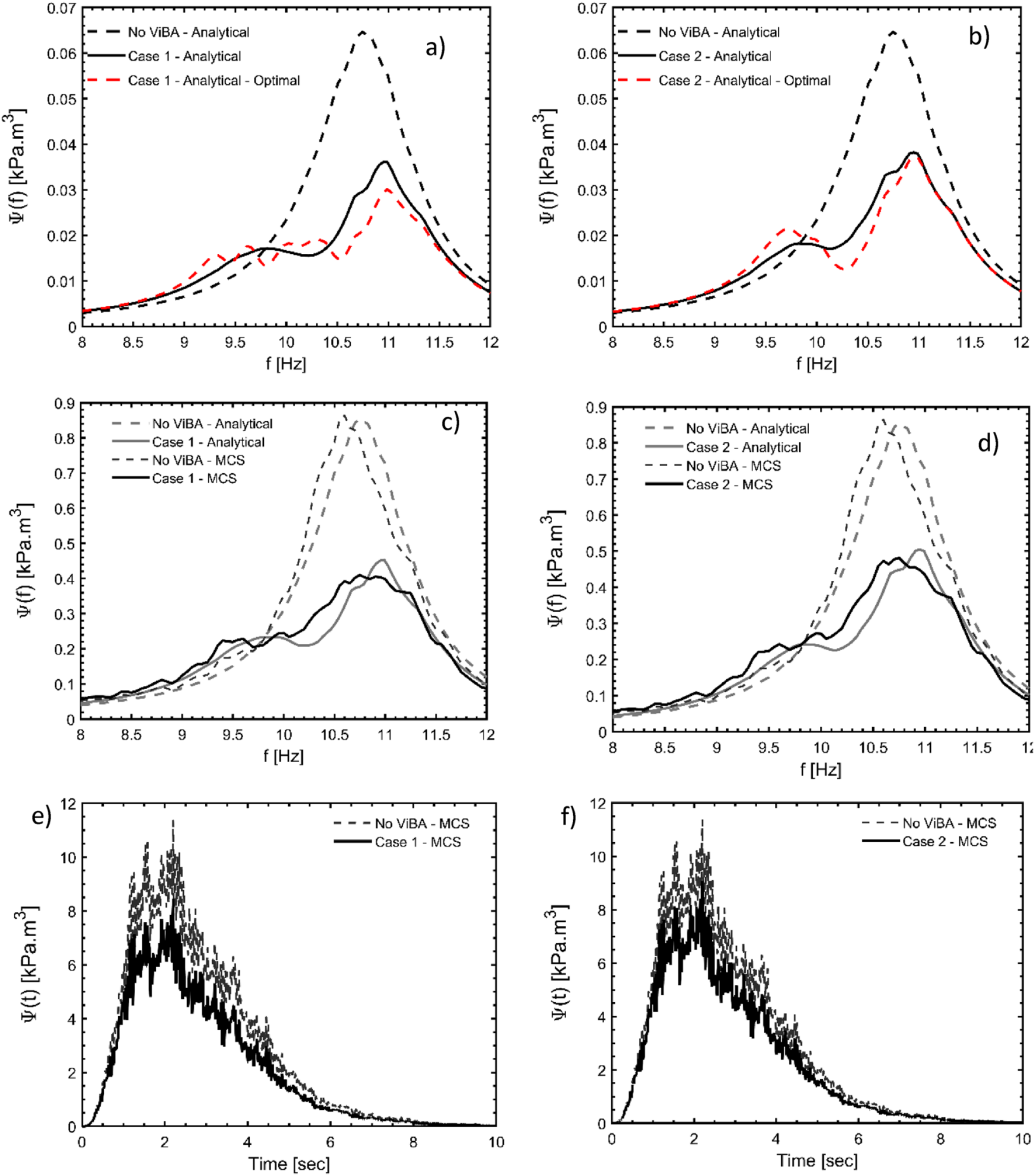
Comparison of the response strain energy of the Pyramid before and after being protected by the ViBa technology. The analytical results have been obtained through Eq. 3 with α;β optimized through the procedure proposed in this paper (and described in the “Method” section) whilst the numerical curves are computed by averaging 500 scaled realizations used for performing a Monte Carlo Simulation (MCS) on the FE model of the Zoser pyramid for the sets of ground motion with Magnitude 5–6. In panel (**a**) the analytical strain energy curves indicated by a dashed line are computed for Case 1 for the scenario of Pyramid without being protected by ViBa (considering its excavation only) and by a dash-red line for the scenario in which the ViBa has been designed with optimal damping, showing a remarkable reduction. Numerical against analytical spectral density strain energy curves are used for verification purposes in panel (**c**). Similar results in panels (**b**) and (**d**) for Case 2. In panels (**e**) and (**f**), the comparison of the strain energy curves in time shows the efficiency of the ViBa for Case 1 and 2, respectively.

**Figure 5:**
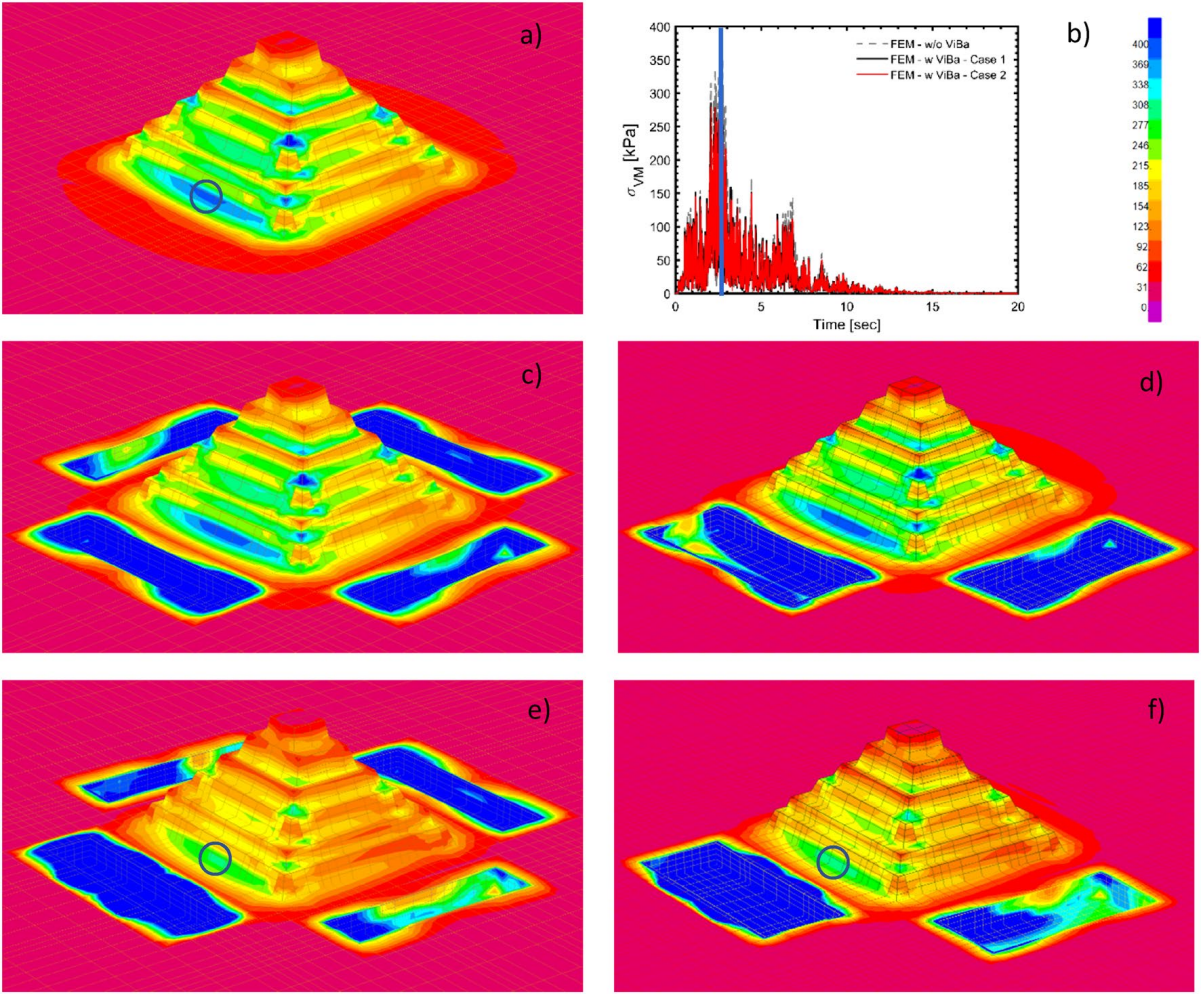
Von Mises stress distribution at t = 2.69 s for a randomly selected simulated ground motion; in panel (**a**) Zoser pyramid without ViBa where it has been highlighted the maximum stress corresponding to the region where the Pyramid experienced damages after the 1992 Cairo earthquake; in panel (**b**) time evolution of the Von Mises stress for this selected element of the Pyramid. Panels (**c**) and (**d**) show the stress distribution considering the trenches only for Case 1 and Case 2; whilst panels (**d**) and (**e**) show the ViBa effect on the stress distribution for Case 1 and Case 2 where it can be observed the sensible reduction of the Von Mises stresses.

**Figure 6:**
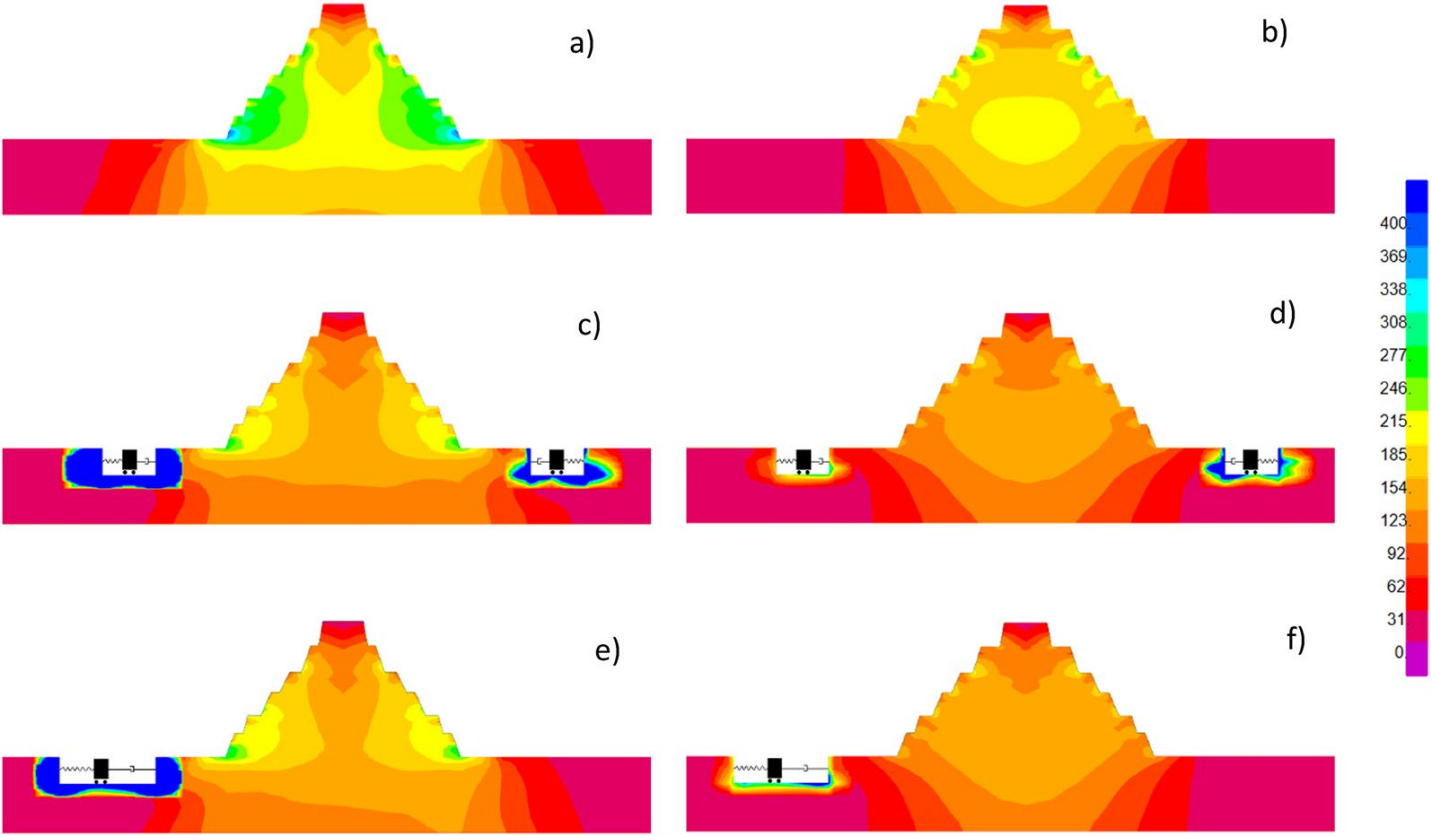
Von Mises stress distribution at t = 2.69 s for a randomly selected simulated ground motion; Panels (**a**) and (**b**) display the Zoser pyramid without ViBa cross section in N–S and E–W direction; Panels (**c**) and (**d**) show the ViBa effect on the stress distribution for Case 1; (**e**) and (**f**) effect of the ViBa on the stress distribution for Case 2.

Firstly, the verification of the proposed design methodology is performed by comparing the analytical versus the average strain energy function for the subset of $M_{0} = \left[ {5 - 6} \right]$; Fig. 4b-c show the good matching computed from the results of the MCS. Then, the strain energy functions in time for Case 1 and Case 2 against the existing scenario are shown in Fig. 4e-f; it is worth mentioning that the curves in frequency and those in time possess the same energy because of the application Parseval’s theorem in the recently proved “strain energy spectral theorem” ^29^. To better display the efficiency of the ViBa technology, results in terms of Von Mises stress are shown in Figs. 5 and 6 for a randomly selected realization and at a specific time instant. Specifically, stress distribution for an individual time history at the time instant of the peak Von Mises stress is reported in Fig. 5a-b. In Fig. 5c-d, the influence of the trenches without the ViBa units is also presented showing the ineffectiveness of the trenches to screen body waves opposite to the ViBa technology.

Figure 6 shows the stress contour maps for the same scenario presented earlier of the N–S and E–W cross sections. Interestingly, the ViBa not only protect the outer surface close to the excavation but is also able to mitigate the stresses in the core of the Pyramid to ensure full protection.

**Figure 7:**
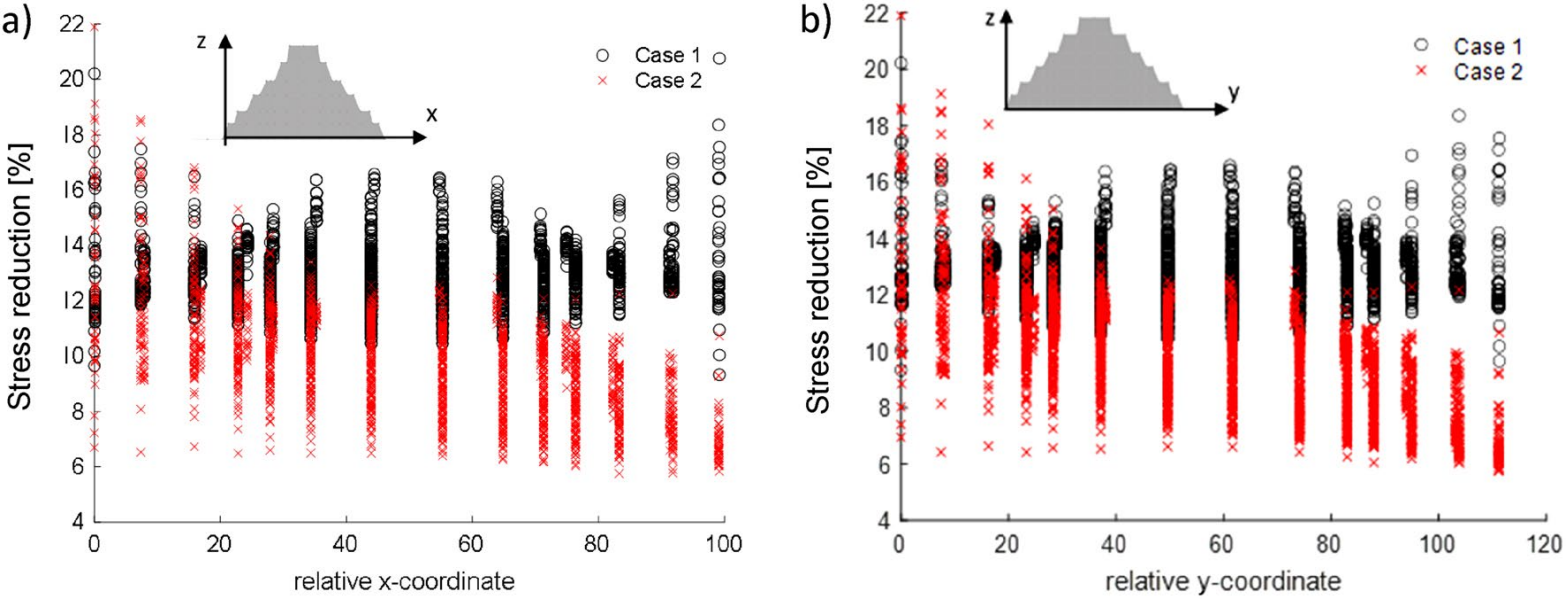
Distribution of the percentage reduction in Von Mises stresses for Case 1 and 2 sorted according to the coordinate of the centroids of each finite element about the (**a**) N–S direction and (**b**) E-W direction. Reductions are higher at the sides where the ViBa units are located; nevertheless, an interesting beneficial effect is observed in the Pyramid’s core.

In Fig. 7, the distribution of the reduction of the peak Von Mises stresses with respect to the existing scenario is plotted for each of the finite element constituting the numerical model according to the 2-principal direction. The efficiency of the ViBa is higher in the proximity of the devices, therefore on both sides for Case 1 and one side for Case 2, with a remarkable maximum reduction of about 22%; nevertheless, reductions can also be observed for every part of the Zoser Pyramid, especially at its core. It is also observed that no negative effects are induced to the Pyramid.

## Experimental tests

**Figure 8:**
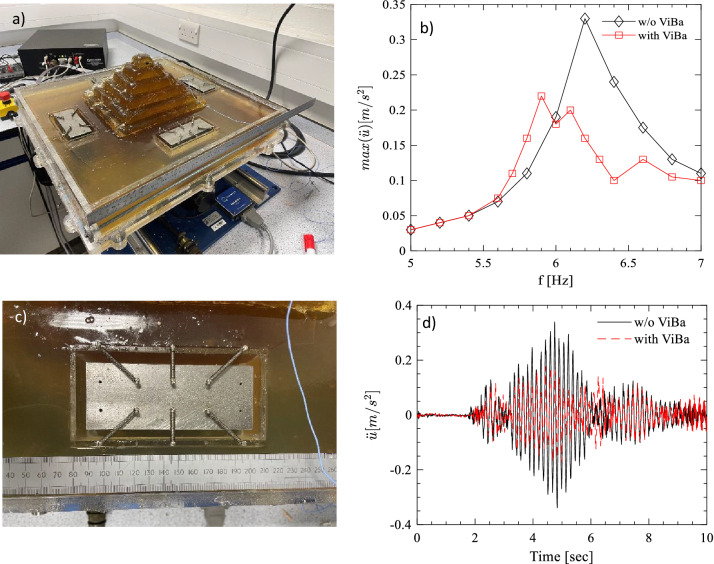
Experimental test on the 1:500 model of the Zoser pyramid: (**a**) test setup; (**b**) frequency response function with and without the ViBas; (**c**) an example of ViBa setup; (**d**) comparison of the acceleration at the top of the pyramid forced by a simulated Cairo earthquake sample (using the Boore^28^ method) showing a reduction of the peak acceleration of about 33% after ViBa protection.

In this section, the effectiveness of the ViBa is also verified experimentally. A reduced scale model (1:500) of the Zoser pyramid and the surrounding area has been built with gelatine (Fig. 8a) and tested on a shake table simulating ground motion excitation.

The Case 1 configuration has been selected for the ViBa design considering a single solid element for each ViBa. The ViBas (Fig. 8b) are made of mild steel bars (114 mm × 44.4 mm × 31.7 mm) of 1.26 kg weight (40% of the mass of the pyramid) and hosted in an acrylic box (134 mm × 68 mm × 39 mm and 4 mm thick) located at 30 mm distance (15 m in the full-scale model) from the base of the pyramid. Each ViBa is supported by two ball bearings to minimize the friction. The tuning frequency is secured by a set of 0.41 N/mm stainless steel springs designed following the procedure presented in “Optimal design of the vibrating barrier” section. The tests have been conducted on Quanser Shake Table II. An accelerometer is mounted on the shake table platform in order to control the accelerations at the base of the test model and a miniature accelerometer is mounted at the top of the pyramid (see Fig. 8a). A series of ground motion harmonic excitation with frequencies ranging from 5 to 7 Hz and acting in the N–S direction have been first performed. In analogy with what has been observed in Fig. 4, the influence of the ViBas on the pyramid response is manifested by the presence of the three peaks (i.e. the first two are corresponding to the ViBas frequency response function peaks while the third one is due to the shift in frequency of the structure) in the frequency response function (rather than one observed for the response without ViBa) and the absorption of part of the kinetic energy in the proximity of the peak response within 6 and 7 Hz (Fig. 8c). For frequencies above 7 Hz, no significant difference has been noted between the cases of ViBa and without ViBa. Moreover, the pyramid has been forced by a scaled accelerogram of the simulated Cairo earthquake with energy spectrum ranging between 0 and 15 Hz. As it can be observed in Fig. 8d a significant reduction of about 33% of the peak response measured at the top of the pyramid has been achieved for the selected ground motion, validating the effectiveness of the ViBa for the seismic protection of the Zoser Pyramid.

## Discussion

In this paper, the non-invasive and non-local vibration control device called Vibrating Barrier has been applied to mitigate the seismic response of the Step Pyramid of Zoser, Egypt. The Pyramid has been already severely damaged during the 1992 Cairo Earthquake event and to guarantee its preservation, the seismic risk should be mitigated to avoid future damages. Because of the lack of available recordings at that time, the seismic activity in the Saqqara’s site relevant to the Zoser Pyramid has been reconstructed through the seismological model introduced by Boore^28^ which parameters have been considered as random variables described by probability distribution functions from available seismological data. The analytical stochastic model has been used to perform the design of the ViBa units as well as to generate acceleration time-history realizations to carry out a Monte Carlo study.

Because of the large numerical model consisting of Pyramid, ViBa units and surrounding soil, a 4-step approach has been proposed to reduce the computational effort during the design stage of the ViBa parameters. The approach proposed sensibly differs from those adopted in previous studies^17^ as it avoids the definition of a reduced-order lumped parameter model for the ViBa design.

Two configurations of seismic protection offered by the ViBa technology denominated Case 1 and Case 2 have been, therefore, tested to assess the efficiency of this novel seismic protection system. Moreover, we propose to adopt for the first time the response strain energy spectral density as objective function of the minimization design problem; this synthetic performance parameter is linked to the peak stresses experienced by the Pyramid and hence, its minimization would lead to meet the aim of this work, namely the seismic protection of the Zoser Pyramid. Results of the Monte Carlo Simulation in terms of peak Von Mises stresses on every element of the Pyramid have shown a significant reduction of the seismic response of the Pyramid when the ViBa device is used. High reductions up to 22% of the seismic response with respect to the existing scenario are obtained on the surfaces closed to the ViBa devices; nevertheless, a beneficial effect is observed in every part of the Pyramid, especially at its core. It is worth mentioning that the highest reductions have been observed in those areas that have been damaged in the past 1992 Cairo Earthquake event. Whilst results have been obtained with a large overall mass of 80% of the Pyramid mass, the analyses conducted for Case 1 and Case 2 showed that smaller masses can be used in several box units without losing the efficiency or by amplifying their inertial forces through inerters^23^. The use of several boxes rather than a single one has certainly few pros (e.g. smaller excavations, versatility to cover multiple frequencies); on the other hand, from a computational point of view, the use of multiple ViBas requires the tedious evaluation of each individual interaction (ViBa-ViBa and ViBa-structure) with also potential ill-conditioning problems. It has also to be emphasized that the structure-soil-structure interaction that is the base of the ViBa working principle can be achieved in alternative ways (e.g. through structures/masses resting on a shallow foundation). This work aimed to show that the ViBa technology is able to protect large heritage structures such as the Zoser pyramid from realistic seismic waves. Specifically, it has been shown that the ViBa was able to protect the Zoser pyramid from horizontal (x and y) ground motion components vertically propagating in the z-direction. In principle, ViBas might also protect the pyramid from the z component, neglected in this paper, by adding vertical spring elements to the device. The dimensions and the typology of excavations the location and the number of the ViBas need to be seen as design requirements used to constrain the optimal ViBa configurations. Therefore, considering the impossibility to use conventional seismic control solutions without affecting the cultural value of heritage structures, the ViBa technology can be seen as a viable solution to guarantee the preservation of the ancient monuments as the Zoser Pyramid.

## Methods

### Finite element analyses

Numerical Finite Element models of the full Pyramid-Soil system are created through the software SAP2000 by using 8-node solid elements. A large soil model with lateral free boundaries, 500 m far away from each edge of the Pyramid, is considered to mitigate the spurious waves reflected back to the system. Fully fixed restraints are used on the bottom of the soil to simulate the rigid bedrock.

### Optimal design: proposed four-stage approach

A reduced-order model capturing the ViBa(s)-Soil-Pyramid interaction is developed to reduce the computational complexity of the optimization problem used to design the ViBa mechanical parameters. Specifically, a sub-structuring method^30^, determined in the frequency domain, is used to condensate the Soil-Pyramid system into complex-valued transfer functions and, to reduce the order of the full system to just 2 translational degrees of freedom per ViBa unit, related to the translational ViBa displacement, $X_{V}$ and to the displacement, $X_{F}$, of its box foundation. Although the design method is developed by considering one ViBa device, the approach can be easily extended for any number of ViBas and any possible configuration and location, by adopting the superposition principle. Therefore, this procedure can be applied rigorously only to linear systems; Nevertheless, because of the high stiffness properties of the soil deposit considered in this study, soil nonlinearites can be neglected in first approximation.

**Figure 9:**
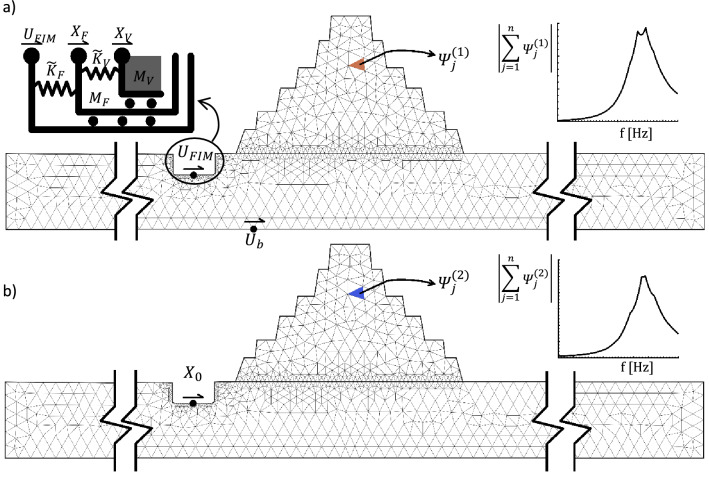
The two sub-domains of the four-step optimal design procedure proposed in this paper. In panel (**a**), the Soil-Pyramid system with the rigid excavation where the ViBa will be installed. The input is applied at the bedrock to derive the interaction between bedrock and foundation input, U_FIM_ as well as the normalized strain energy spectral density of the Pyramid. A close-up of the reduced-order mechanical system used to model the ViBa unit is also shown. In panel (**b**), the second sub-domain is depicted. The input is applied to the centre of rigidity of the foundation (X_0_) to determine the effect of the ViBa box foundation on the Pyramid dynamic response.

The proposed method consists of subdividing the full domain into 2 partitions: the first subdomain represents the Soil-Pyramid system with any rigid excavations where the ViBas will be installed (Fig. 9a); the second subdomain comprises the internal structure of the ViBa contained by the rigid box-foundation resting on a flexible soil medium modelled through soil-foundation impedance functions (Fig. 9b). A four-step approach is devised as follows: (i) a steady-state analysis of the first subdomain subjected to bedrock ground motion displacement, $U_{b} \left( f \right)$, modelled as unitary constant function to derive the foundation input motion of the rigid excavation, $U_{FIM}^{{}} \left( f \right)$, as well as the normalized response strain energy spectral density of the Pyramid, ${\Psi }_{{}}^{\left( 1 \right)} \left( f \right)$; (ii) a steady-state analysis of the first subdomain where a unitary displacement, $X_{0} \left( f \right)$, is applied to the centre of the rigidity of the excavation (Fig. 9b) to derive the response strain energy spectral density of the Pyramid, $\Psi _{{}}^{{\left( 2 \right)}} \left( f \right)$, as well as the complex-valued soil-foundation impedance, $\tilde{K}_{F} \left( f \right)$; (iii) an inertial analysis of the ViBa-Soil subdomain where the soil is modelled through the impedance $\tilde{K}_{F} \left( f \right)$; and iv) recovery of the complete response strain energy spectral density of the Pyramid by adopting the superposition principle.

After computing the various complex-valued energy functions, ${\Psi }_{{}}^{\left( 1 \right)} \left( f \right)$ in step (i) and $\Psi _{{}}^{{\left( 2 \right)}} \left( f \right)$ in step (ii), the design of the optimal ViBa parameters is performed on the reduced-order model of step (iii) and (iv) in which an extremely fast optimization analysis has been implemented. Considering the mechanical system of Fig. 9a, the governing equations of the reduced-order model can be written in the frequency domain as follows:

4 $$\left\{ {\begin{array}{*{20}l} {\tilde{K}_{V} X_{V} - \tilde{K}_{V} X_{{\text{F}}} - \omega^{2} M_{V} X_{V} = 0 } \\ {(\tilde{K}_{F} + \tilde{K}_{V} )X_{{\text{F}}} - \tilde{K}_{V} X_{V} - \omega^{2} M_{F} X_{{\text{F}}} = \tilde{K}_{F} U_{FIM}^{{}} } \\ \end{array} } \right.$$

where the dependencies from the frequency $f$ have been omitted. Equation (4) represents a system of algebraic equations in which $X_{V}$ and $X_{{\text{F}}}$, are the unknown ViBa’s displacement and foundation displacement, respectively, whilst $\tilde{K}_{V}$ is the ViBa parameter to be optimized. To perform the optimal design of the ViBa, the complete response strain energy spectral density ${\Psi }\left( {{\varvec{\alpha}};{\varvec{\beta}};{f}} \right)$ of the Pyramid is computed and the optimal parameters of the ViBa are determined through the following minimization problem using the Simplex search algorithm:

5 $$\mathop {\min }\limits_{{{\varvec{\beta}} > 0}} \left( {\mathop \int \limits_{0}^{\infty } \mathop \int \limits_{{A}}^{{}} {\Psi }\left( {{\varvec{\alpha}};{\varvec{\beta}};{f}} \right)p_{{A}} \left( {\varvec{\alpha}} \right){\text{ d}}{\varvec{\alpha}}{\text{df}}} \right)$$

### Experiment set up

The model has been made of a mix of gelatine/glycerine/cold water/hot water with weight proportions 1/3/2/3 and cured for 3 days. The pyramid mould has been done with silicone rubber, while an acrylic box has been used for the mould of the area around the pyramid and to facilitate the connection with the shake table. Preliminary modal tests have been conducted to identify the material properties of the gelatine (modulus of elasticity $E_{gelatine} = 21.6{\text{ kPa}}$, Poisson's ratio, $\upsilon_{gelatine} = 0.49$,.density $\rho_{gelatine}$, of 1078 ${\text{kg}}/{\text{m}}^{3}$, modal damping $\zeta_{gelatine}$ = 0.058) as well as the damping of the ViBa ($\zeta_{ViBa}$ = 0.02). Quanser Shake Table II (46 × 46 cm, operational bandwidth 0-20 Hz, peak acceleration 2.5 g) has been used for simulating the ground motion. Pyramid accelerations have been measured through the miniature lightweight (0.2 g) accelerometer (ceramic shear ICP^®^ accel., 5 mV/g, 2 to 10 k Hz) and the LMS SCADAS mobile acquisition system.
